# The first record of Brachycyrtinae (Hymenoptera, Ichneumonidae) in Finland

**DOI:** 10.3897/BDJ.13.e161537

**Published:** 2025-07-24

**Authors:** Emil M. Österman, Max M. J. Koistinen, Kari M. Kaunisto, Iida A. E. Österman, Anssi A. Teräs, Juulia Räikkönen, Ilari E. Sääksjärvi

**Affiliations:** 1 Biodiversity Unit, University of Turku, Turku, Finland Biodiversity Unit, University of Turku Turku Finland; 2 Department of Biology, University of Turku, Turku, Finland Department of Biology, University of Turku Turku Finland; 3 Biological Collections of Åbo Akademi University, Zoological Museum, University of Turku, Turku, Finland Biological Collections of Åbo Akademi University, Zoological Museum, University of Turku Turku Finland

**Keywords:** biodiversity, *Brachycyrtusornatus*, distribution, morphological variation, Skanssi Biodiversity Park, Turku, urban biodiversity

## Abstract

**Background:**

Brachycyrtinae is a small, but distinctive subfamily of Darwin wasps (Hymenoptera, Ichneumonidae) with one widespead species in Europe: *Brachycyrtusornatus* Kriechbaumer, 1880. We recently collected the species during the faunistic survey of the Urban Biodiversity Parks project in Skanssi, Turku, south-western Finland.

**New information:**

We report the Darwin wasp *Brachycyrtusornatus* as the first record of the subfamily for Finland. We collected a single female specimen with a Malaise trap in Skanssi Biodiversity Park during a period from August to September 2024. This is the northernmost record of *B.ornatus* and the subfamily Brachycyrtinae as a whole. Notes on the species' distribution and morphological variation are included.

## Introduction

Brachycyrtinae is a small, rarely collected subfamily of Darwin wasps (Hymenoptera, Ichneumonidae) with a single genus: *Brachycyrtus* Kriechbaumer, 1880 (Wahl and Gauld 2002). Currently, 27 species of the genus have been described (Broad et al. 2018, Di Giovanni and Varga 2021, Nascimento and Fernandes 2024). It has a cosmopolitan distribution, with records from all continents other than Antarctica (GBIF 2025a), although most species are tropical.

Members of the genus *Brachycyrtus* can easily be distinguished from other Darwin wasps by their hunched mesosoma, fore-wing vein *cu-a* being separated from *Cu* by more than half the length of *cu-a* and their distinctive yellow-black or yellow-black-orange colouration (Broad et al. 2018). Biological information is scarce for the genus, but at least some species are known as solitary parasitoids of green lacewing (Neuroptera, Chrysopidae) cocooned prepupae and pupae (Gauld and Ward 2000). Their phylogenetic location and morphology suggest they are idiobiont ectoparasitoids (Short 1978, Wahl 1993).

A single holarctic species is known from Europe: *Brachycyrtusornatus* Kriechbaumer, 1880. It has the northernmost distribution of all species of the genus, having been recently recorded as far north as in southern Sweden and Norway (GBIF 2025b).

Here, we report a specimen of *Brachycyrtusornatus*, recently collected in Turku, south-western Finland, which represents the first record of the subfamily for Finland. Notes on the distribution and morphological variation of the species are included. This is also amongst the first published findings of the ongoing survey of the Urban Biodiversity Parks project in Skanssi, Turku, which trials and develops the concept as a co-creative platform for enhancing biodiversity, learning and community involvement in urban ecological regeneration (European Urban Initiative 2025).

## Materials and methods

**Figure 1.**
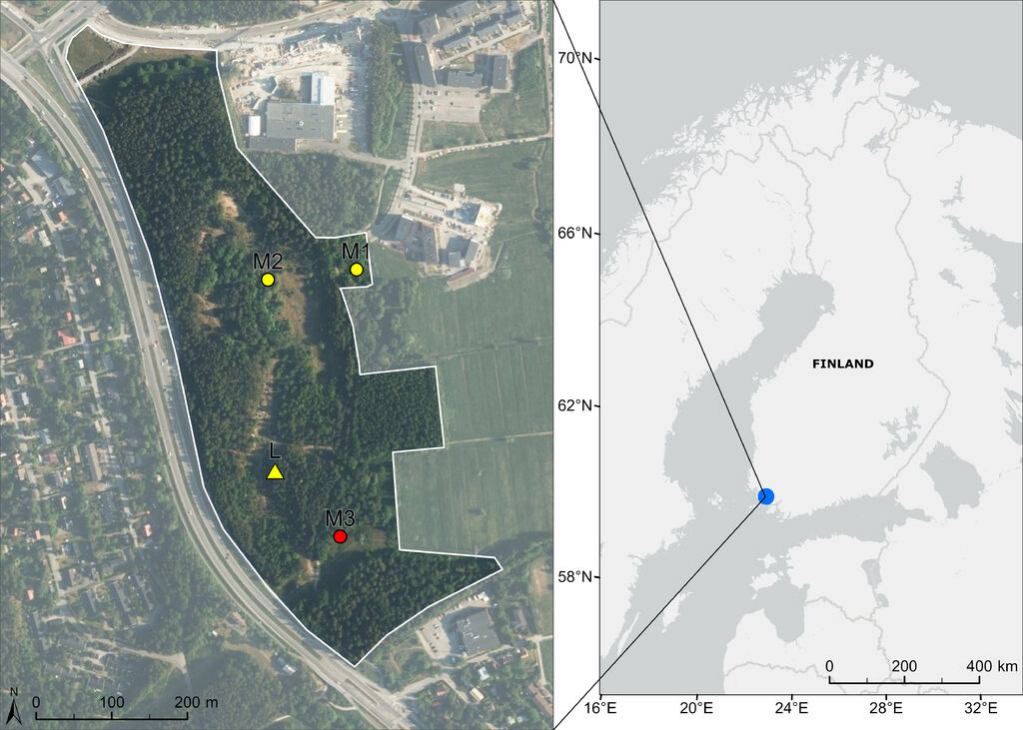
Map of the study area (left; Skanssi Biodiversity Park, Turku) and its location in Finland (right; blue circle). Yellow and red figures: circle (M) = Malaise trap (M3 in red is the collecting trap of *Brachycyrtusornatus*); triangle (L) = light trap. The aerial photograph of the study area was obtained from NLS orthophotos, National Land Survey of Finland (07/2023), licence CCBY-4.0.

The study area, Skanssi Biodiversity Park (60.4238°N, 22.3183°E), is a 20 ha urban park in Turku, south-western Finland (Fig. 1). We conducted faunistic surveys, targeting primarily insects, using various sampling methods: line transect surveys, light traps, bait traps, Malaise traps and pitfall traps. All traps were operational throughout the sampling season (23 May 2024–30 Oct 2024) to ensure comprehensive coverage of insects.

**Figure 2.**
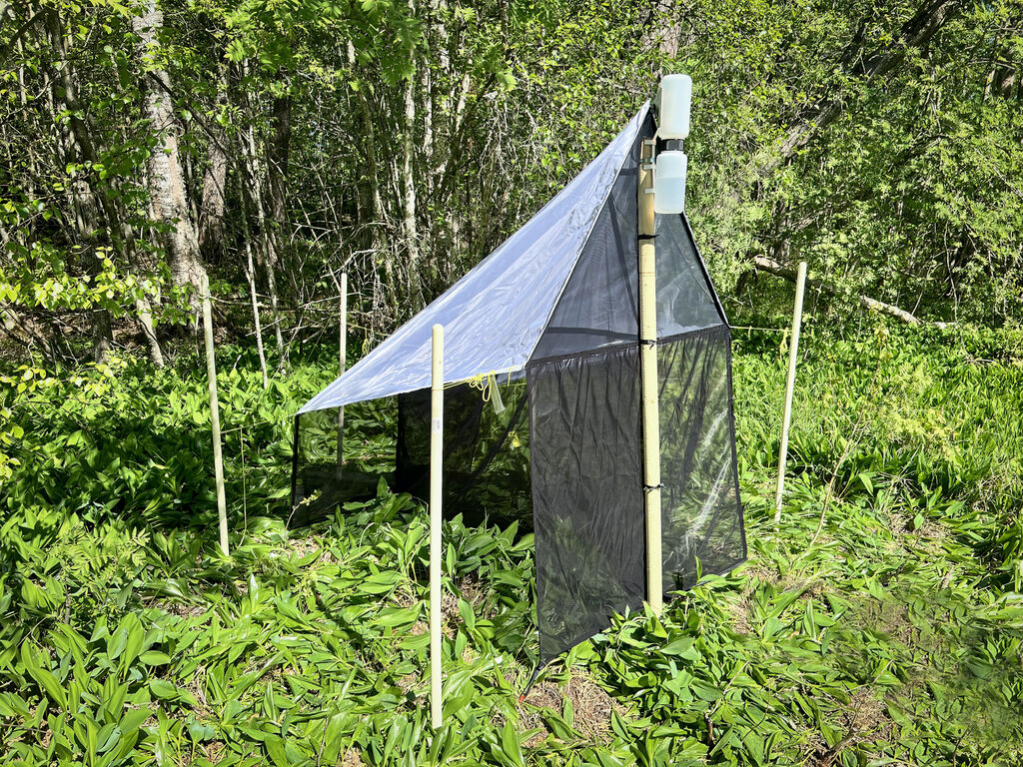
Malaise trap (M3) in Skanssi Biodiversity Park, Turku, Finland.

We sampled the Darwin wasp fauna using three Malaise traps (15.9 trap months) and one light trap (4.1 trap months) placed in different habitats (Fig. 1). Malaise traps are tent-like structures of fine mesh that guide flying insects upwards into a collecting chamber with lethal perservative, here 70% ethanol (emptied monthly). The traps were a standard size (Fig. 2), manufactured by Watkins and Doncaster (Leominster, Herefordshire, UK).

We separated all Darwin wasps from collected Malaise and light trap samples and deposited them at ZMUT (the Zoological Museum of the University of Turku, Finland). The samples contained one *Brachycyrtusornatus* specimen, collected by a Malaise trap (M3 in Figs 1, 2) positioned at the edge of a powerline clearing. The trap faced the clearing, which featured a plant community dominated by red raspberry (*Rubusidaeus*), false spiraea (*Sorbariasorbifolia*), alder buckthorn (*Frangulaalnus*) and red elderberry (*Sambucusracemosa*). The ground layer near the trap was mainly covered by lily of the valley (*Convallariamajalis*). The rear of the trap faced broadleaf forest.

We photographed the specimen using a Sony Alpha 9 Mark II camera body mounted on a macro-rail, equipped with an extension tube, a relay lens and Mitutoyo Plan Apo objectives with magnifications ranging from 2.5× to 20×, which enabled us to control and incrementally move the camera between shots. We captured multiple images at successive focal depths and combined them using Helicon Focus software to produce composite layer images with extended depth of field. We carried out final image adjustments in Adobe Photoshop CC to ensure accurate representation of the specimen's morphological features. Morphological terminology follows Gauld (1991).

## Taxon treatments

### Brachycyrtus ornatus

Kriechbaumer, 1880

E6150D4A-BD59-58B2-AB48-C4073EB9A211

#### Materials

- **Type status:** Other material. **Occurrence:** recordedBy: Max M. J. Koistinen, Anssi A. Teräs; individualID: ZMUT.35504; individualCount: 1; sex: female; occurrenceID: 35161C25-6E24-511D-B7A9-2A2313E9D092; **Taxon:** scientificName: *Brachycyrtusornatus*; **Location:** country: Finland; municipality: Turku; locality: Skanssi Biodiversity Park; locationRemarks: edge of powerline clearing next to forest; verbatimCoordinates: 60.421141°N, 22.320628°E; verbatimSRS: WGS84; **Identification:** identifiedBy: Emil M. Österman, Ilari E. Sääksjärvi; **Event:** samplingProtocol: Malaise trap; eventDate: 6 Aug 2024–20 Sep 2024; **Record Level:** institutionCode: ZMUT; basisOfRecord: PreservedSpecimen; source: http://mus.utu.fi/ZMUT.35504

#### Diagnosis

This species can easily be separated from its single congener with an overlapping range in the Nearctic (*B.pretiosus* Cushman, 1936) by the following characteristics: face variably darkened; propodeum with area petiolaris and area superomedia confluent (i.e. transverse carina in the middle of the propodeum is absent); mesoscutal setae about the same length as metapleural and propodeal setae (modified from Wahl and Gauld (2002)).

#### Distribution

Finland (new record); widespread in the Holarctic.

#### Morphological remarks

**Figure 3.**
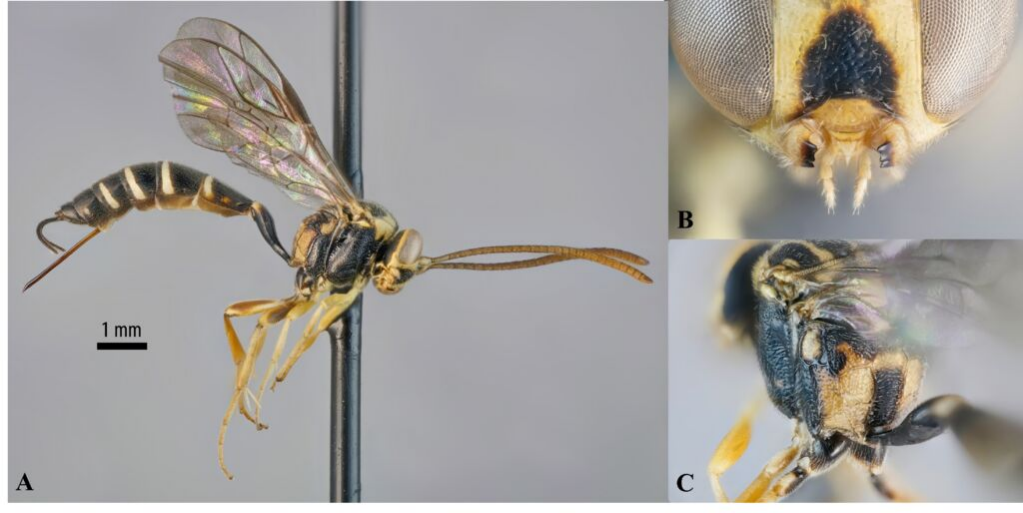
*Brachycyrtusornatus*, female. **A** Habitus; **B** Mandibles; **C** Propodeum.

The dorsal tooth of this species has, like some of its congeners (Wahl and Gauld 2002), an impressed apex (Fig. 3), making the mandible appear tridentate (similar to Diplazontinae). In common with other species of *Brachycyrtus*, many carinae are unusually well developed amongst Darwin wasps, see, for example, the nearly complete propodeal carinae and the epomia (Fig. 3).

## Discussion

### Distribution

This finding represents the northernmost record of *Brachycyrtusornatus* and Brachycyrtinae as a whole, although not by much, as the *B.ornatus* records from southern Sweden and Norway are latitudinally close (GBIF 2025b). This species is likely a recent colonist in south-western continental Finland, as ichneumonologist Reijo Jussila has researched Darwin wasps in the region for many decades without encounters (R. Jussila, pers. comm.). Moreover, the *B.ornatus* records from Sweden and Norway show a tentative trend of records at higher latitudes being more recent than records at lower latitudes (GBIF 2025b). Therefore, it is likely that the northern edge of this species' range is expanding, possibly driven by global climate warming. This is, however, uncertain as Darwin wasps are understudied insects and Brachycyrtinae are rarely collected (Wahl and Gauld 2002). In the Palaearctic, *B.ornatus* has been recorded as far south as in France, Italy, North Macedonia, Turkey and Iran (Kolarov and Ghahari 2007, Kolarov 2009, Çoruh et al. 2016, GBIF 2025b). Based on a range estimation of the European records, the species likely occurs in many countries currently without records, for example, the Baltic countries.

### Morphological variation

Besides obvious sexual dimorphism in the morphology of the structures of the latter metasomal tergites, *B.ornatus* males and females have been noted to otherwise be similar (Karlsson and Magnusson 2011). However, based on some specimens in Sweden, many corresponding areas with yellow patches in the females are absent or underdeveloped in the males (replaced by black; Karlsson and Magnusson (2011)). There is significant colour variation in females, particularly in the size of the yellow patches and the amount of reddish colour on the metasomal tergites, for example, some females can have a uniformly black propodeum (see e.g. Vas (2013)), whereas the propodeum of our specimen bears a large, inverted U-shaped, yellow figure (Fig. 3); some specimens from Italy can be described as yellow with black patches and an entirely yellow propodeum (F. Di Giovanni, pers. comm.). Metasomal tergites may contain large red portions, usually on tergites 2–3 (see specimens in GBIF (2025b)), which are rarely indicated in males (F. Di Giovanni, pers. comm.) or be more or less entirely black, except for the yellow hind margin (see, for example, Vas (2013) or Fig. 3). Consistent geographical patterns of colour variation are not evident based on the current available photographs of the species from different locations.

### Skanssi Biodiversity Park

At the outset of the Urban Biodiversity Parks project, the Zoological Museum of the University of Turku (Biodiversity Unit) conducted a comprehensive faunistic, floristic and soil survey. This baseline dataset serves several important functions. It provides a detailed overview of species diversity for relevant stakeholders, establishes a reference point for long-term monitoring of ecological restoration efforts and supports future evaluations of restoration outcomes at ten-year and twenty-year intervals. Additionally, the survey contributes to the development of urban biodiversity conservation strategies through the application of innovative approaches, including environmental DNA (eDNA) analysis used in soil surveys.

Our discovery of *Brachycyrtusornatus* in Finland, a result of the project's faunistic survey, highlights that even urban or semi-urban areas can host rare or previously unrecorded species. These can be discovered in surveys and may be incorporated into strategies aiming to increase biodiversity as subject species of long-term monitoring. In the context of the Urban Biodiversity Parks project, such findings may also promote increased community engagement and interest in local biodiversity strategies and outcomes.

## Supplementary Material

XML Treatment for Brachycyrtus ornatus
